# Effects of Mobile Electromagnetic Exposure on Brain Oscillations and Cortical Excitability: Scoping Review

**DOI:** 10.3390/s25092749

**Published:** 2025-04-26

**Authors:** Azadeh Torkan, Maryam Zoghi, Negin Foroughimehr, Ali Yavari, Shapour Jaberzadeh

**Affiliations:** 1 Monash Neuromodulation Research Unit, Department of Physiotherapy, School of Primary and Allied Health Care, Monash University, Melbourne, VIC 3199, Australia; shapour.jaberzadeh@monash.edu; 2 Discipline of Physiotherapy, Institute of Health and Wellbeing, Federation University, Melbourne, VIC 3842, Australia; m.zoghi@federation.edu.au; 3 Australian Synchrotron, Australian Nuclear Science & Technology Organisation, Melbourne, VIC 3168, Australia; nforoughimehr@swin.edu.au; 4 6G Research and Innovation Lab, Swinburne University of Technology, John Street, Hawthorn, Melbourne, VIC 3122, Australia; ayavari@swin.edu.au

**Keywords:** electromagnetic exposure, brain oscillations, electroencephalography, corticospinal excitability, 5G, EME

## Abstract

With the widespread adoption of smartphones, concerns about increased exposure to non-ionizing radiofrequency have emerged. This scoping review examines the effects of mobile phone exposure on neural oscillations and cortical excitability, focusing on both motor and non-motor regions of the cerebral cortex. A scoping review identified seventy-eight studies that involved healthy individuals and employed electroencephalography and only two studies that investigated transcranial magnetic stimulation as primary technical tools. The findings suggest that mobile phone exposure may affect brain oscillations and cortical excitability. However, inconsistencies in experimental methods across studies make it difficult to draw definitive conclusions. Additionally, research on fifth-generation technology, particularly mmWave exposure from next-generation mobile networks, remains limited and needs further exploration. These gaps highlight the need for more in-depth studies on how mobile phone exposure impacts brain function.

## 1. Introduction

Mobile phones are compact devices with telecommunication and computational capabilities, which can easily fit in a pocket and are seamlessly integrated into every aspect of daily life. They serve a wide range of functions, including communication, video recording, viewing, photography, and more, offering diverse solutions through various applications.

Driven by the need for high-speed, reliable and accessible communication for time-sensitive, data-driven, and large-scale innovation and technologies such as the Internet of Things, the mobile industry is constantly evolving [1,2,3]. Over the years, five generations of mobile networks have been developed, which mostly operated at frequencies below 6 gigahertz (GHz). Each generation represents a remarkable advancement in mobile technology and introduces faster data speeds and enhanced features, which revolutionize the way we connect and communicate. The current and fifth generation (5G) represents a major shift; it uses higher frequencies to support the increasing demands of applications like high-resolution video streaming, telepresence, and virtual reality (VR).

Regarding 5G technology, it delivers data transfer speeds of up to 10 gigabits per second (Gb/s) with latency under one millisecond (ms), which was achieved through advancements in mobile technologies and the utilization of millimeter-wave (mmWave) frequency bands (e.g., 26 GHz and higher), thus expanding spectrum usage beyond the traditional sub-6 GHz range. Initially defined as the sub-6 GHz frequency bands (450–6000 megahertz (MHz)), Frequency Range 1 (FR1) has now expanded to cover 410–7125MHz, commonly referred to as the mid-band/low-band. FR1 has been utilized in mobile networks since the first generation nearly four decades ago, with extensive research conducted on its exposure effects. Many countries are preparing to deploy 5G spectrum in Frequency Range 2 (FR2) spanning 24,250–52,600 MHz; this shift aims to support a wide range of applications [4].

Electromagnetic (EM) waves are crucial for enabling wireless communication and are classified as either ionizing or non-ionizing. These waves are generated by repetitive, synchronized vibrations of electric and magnetic fields and propagate through space at $3\times 10^{8}$ m per second, transferring radiant energy [5]. The EM spectrum is divided into radio waves, microwaves, infrared, visible light, ultraviolet, X-rays, and gamma rays [6].

Non-ionizing exposure, which includes UV, visible light, infrared, microwave, and radiofrequency (RF) waves, does not possess sufficient energy to initiate the ionizing process [7,8]. RF exposure consists of electromagnetic (EM) waves with frequencies ranging from 100 kilohertz (kHz) to 300 GHz. Several factors influence RF exposure, such as the type of wave (continuous or pulsed), electric and magnetic field strengths, power density, exposure type and zone, source frequency, and exposure duration [9]. The dielectric properties of water in the 6–300 GHz range result in an absorption coefficient (at 20 °C), which increases from 4 cm^−1^ at 6 GHz to 30 cm^−1^ at 30 GHz, 85 cm^−1^ at 100 GHz, and 150 cm^−1^ at 300 GHz [4]. Consequently, the penetration depth is approximately 1 mm at 30 GHz and decreases to around 0.3 mm at 100 GHz, which indicates that EM energy is primarily absorbed in the superficial layers of tissues at higher frequencies. A significant question regarding public health is how EM exposure will vary as mobile telecommunications transition to higher-frequency technologies, including those for millimeter-wave (mmWave) frequencies, compared to Frequency Range 1 (FR1).

Research on the effects of mobile phone EM exposure is essential for understanding how RF-EMFs influence brain function, especially brain oscillations (rhythmic electrical patterns) and cortical excitability (CSE), both of which are vital for cognition, motor control, and sensory processing. Since mobile phones emit RF-EMFs and are often used near the head for long periods, there is growing concern about the potential impact of long-term daily exposure on cognitive and visuomotor tasks [10] and sensorimotor pathways involved in Gait and Balance Control [11], and reaction time [12,13]. In addition, prolonged mobile phone EM exposure has been linked to subjective reports of localized discomfort, such as headache, dull pain, or weakness near the ear [14,15,16,17,18]. While the mechanisms remain unclear, hypotheses include thermal effects on superficial tissues or indirect activation of pain receptors by mmWave penetration in 5G frequencies [4,7].

New studies are emerging to assess the impact of higher-frequency millimeter waves (used in 5G) on brain tissue. Since these waves have lower penetration depths, the focus has shifted to superficial tissues, but concerns about cumulative exposure and indirect brain effects remain. The limited number of studies in this area highlights a clear gap in the literature.

Currently, there is no agreement on the effects of mobile EM exposure on brain activity. While some findings suggest that mobile EM exposure leads to increased brain activity within specific frequency ranges, others demonstrate a reduction in specific frequency bands. For example, experiments in [19] have shown that EM exposure in the 900–1800 MHz range results in a decrease in delta band activity and increased amplitudes in the alpha, beta, and theta bands. Additionally, another study revealed that mobile EM exposure in the 900 MHz range causes a decrease in alpha band power during exposure sessions in eyes closed conditions, with no effect in eyes opened conditions [20].

Similarly, different studies have reported conflicting results on the effect of mobile EM exposure on brain excitability. For instance, exposure to 902.40 MHz mobile phone EM has been found to significantly increase intracortical facilitation (ICF) while decreasing short intracortical inhibition (SICI) in comparison to the non-exposed hemisphere or sham exposure [21]. However, another study [22] reported that exposure to low-frequency EM, such as 800 MHz, cannot induce changes in CSE and SICI.

This scoping review, unlike a systematic review, summarizes existing research on RF-EMF effects (2G–4G) on brain oscillations and CSE on both motor and non-motor cortical regions without generating new experimental or numerical data. It highlights the lack of study on how 5G mobile phone EM exposure may alter neural activity. Therefore, this review focuses on addressing the main research question: What are the effects of mobile phone EM exposure on brain oscillations and cortical excitability in healthy individuals, and what gaps exist in the current understanding of these effects?

Through a mapping of the literature, this review verifies that its primary aims are simultaneously fulfilled by the EEG and TMS studies:

1. To map existing literature and evaluate the effect of mobile EM exposure on changes in EEG band power amplitudes in eyes opened or eye closed conditions.
2. To map existing TMS literature and evaluate the effects of mobile EM exposure on changes in CSE and the underlying mechanisms, such as SICI, LICI, and ICF.
3. To identify literature gaps and propose suggestions for future studies.

## 2. Materials and Methods

A scoping review selection was conducted using the following online databases: MEDLINE (https://www.nlm.nih.gov/medline, accessed on 1 July 2024), PubMed (https://pubmed.ncbi.nlm.nih.gov, accessed on 1 July 2024), Web of Science (https://www.webofscience.com, accessed on 1 July 2024), and Scopus (https://www.scopus.com, accessed on 1 July 2024).

In this review, only English-language studies were considered. The search was conducted from the 1980s of each included database until July 2024. The title, abstract, and keyword fields were screened. The search terms included in Table 1 were combined using ‘OR’ and ‘AND’ to complete the search.

The participants for this review are healthy individuals, aged 18 to 65, without any neurological, psychological, or musculoskeletal conditions. Animal studies or studies on patients have been excluded.

The included studies comprised primary research studies, randomized controlled trials (RCTs), case (sham) control studies, case-control crossover studies, case series, systematic reviews, meta-analyses, and narrative reviews that investigated the effects of mobile EM exposure on neural oscillations or cortical excitability. Conference reports were excluded to prevent data duplication.

### 2.1. Methodological Approach

This review was conducted following the guidelines of the Preferred Reporting Items for Scoping Review (PRISMA) guidelines [23,24].

**Figure 1:**
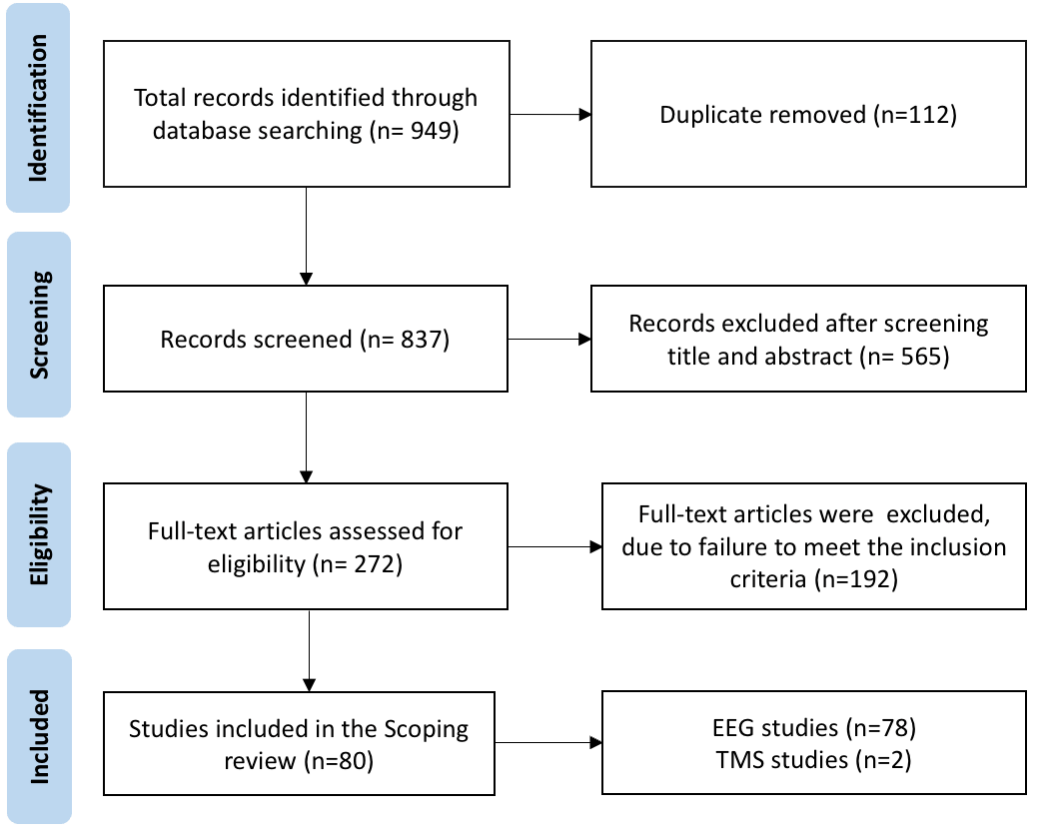
Flow diagram showing the selection process of articles following PRISMA guidelines.

The obtained results were managed using EndNote X9 (Clarivate Analytics, Philadelphia, PA, USA), where duplicate papers were removed for further review and screening (see Figure 1).

The remaining papers were screened separately for their eligibility following the exclusion of duplicates. Furthermore, the titles and abstracts of the studies were evaluated, leading to the exclusion of those that did not meet the inclusion criteria. Subsequently, the full texts of the remaining studies underwent scrutiny based on determined inclusion and exclusion parameters.

### 2.2. Extraction of Data

The subsequent information was extracted and organized in a tabular format (Table 2, Table 3 and Table 4) for every study that met the inclusion criteria. The emphasis of the included studies focused on the energy of all mobile phone technologies, 3G, 4G, and 5G, that affect the brain with a specific focus on brain oscillation and CSE, which are objectives of this review. This review approach aligns with PRISMA’s emphasis on detailed and transparent data reporting.

Table 2 and Table 3 present a structured summary of the review outcomes, featuring details such as authorship, publication year, participant specifics (number, gender, and age range), mobile EM exposure parameters (frequency range and power density, Specific absorption rate (SAR)), duration of exposure, blinding/study design, EEG attributes (number of channels and electrode configuration), and recorded encephalogram details such as if the EEG recording is in the eyes open or eyes close condition and finally the mentioned impact on all band power amplitude which is the one of the target of this review. Moreover, these tables provide insights into the primary findings and their corresponding effects. Table 4 is organized similarly to the previous tables, including all parameters, but evaluates the effect of mobile EM exposure on CSE and Corticocortical Excitability (CCE).

Quantitative electroencephalography (qEEG) was employed to record and evaluate brain oscillations before and after exposure to mobile EM energy. EEG signals were evaluated using two outcome measures: 1. EEG band power amplitude, including delta (0.5–4 Hz), theta (4–8 Hz), alpha (8–13 Hz), beta (13–30 Hz), and gamma (30–90 Hz) bands [96,97,98], and 2. connectivity, which signifies correlated signals between functionally related brain regions [99].

TMS was used to assess two outcome measures pre- and post-mobile phone exposure: 1. the peak-to-peak amplitude of single-pulse TMS-induced MEPs for assessment of CSE, and 2. the paired-pulse TMS paradigm for assessing CCE, including SICI [100], LICI [101] and ICF [102].

## 3. Results

A total of 949 studies were obtained through various databases. Among these, 112 articles were singled out, following the exclusion of duplicates based on the screening of titles and abstracts (Figure 1). A total of 565 articles were excluded after the screening, and 192 papers were excluded in the next step, based on eligibility criteria, resulting in 80 articles that were incorporated into this review. Among these, seventy-eight papers pertained to EEG studies, while two papers focused on TMS studies. The initial search strategy that will be used when searching the MEDLINE or (PubMed) database is presented in Figure 1.

We followed the PRISMA guidelines, which outline the essential components for systematic and scoping reviews and evidence-based meta-analyses [24,103]. We methodically examined relevant literature concerning the effects of mobile EM exposure on the brain within the context of a scoping review.

### 3.1. Effects of Mobile EM Exposure on Brain Oscillations

A total of 80 papers were included in this review. Out of this, 78 studies are EEG studies and focused on the effects of mobile EM exposure on brain oscillations. Brain oscillations, known as brainwaves, refer to rhythmic patterns of neural activity within the central nervous system. Neural tissue can produce oscillatory activity through various mechanisms, including processes within individual neurons or interactions among multiple neurons from different brain regions [104]. The emission of EMR via mobile phones can lead to alterations in these brain activities. Most of the studies, specifically 78 out of 80 (97.5%), that focused on investigating the effects of mobile EM exposure on band power amplitude indicated that mobile EM exposure led to various alterations in brain oscillations, encompassing both increases and decreases. However, a smaller number of studies referred to (Table 2 and Table 3), yielded inconclusive results or demonstrated no evident effects.

A remarkable finding [85] indicates changes in alpha band amplitude, both increasing and decreasing, occurred only under specific electrodes. Generally, following the exposure to EMR, the presented studies reported various results, both an increase and a decrease in all EEG band amplitudes in EEG recordings under different conditions such as eyes opened or eyes closed; others yielded inconclusive results or demonstrated no evident effects.

The data have been divided into two tables: Table 2 presents data for eyes opened, and Table 3 presents data for eyes closed. This categorization was implemented to improve clarity, facilitate clearer visualization, simplify the information for presentation, and enhance comparability. This format supports researchers in developing more precise theories, research questions, and aims based on the observed effects in each condition, guiding future studies and experimental designs. Moreover, separate tables facilitate an easier understanding of the effects of mobile EM exposure on different EEG bands’ power, allowing readers to draw conclusions and recognize consistent outcomes. The observed results are summarized in Table 2 and Table 3.

Some studies examined both conditions within the same experiment, presenting different results across both tables [36,37]. In this scoping review, as is conventional in EEG recording, the absence of mention regarding the condition of the eyes during the experiment is typically interpreted to mean that the eyes were open. Notably, in the selected papers, the predominant findings indicate increased band power amplitude, while the key independent variables include mobile EM exposure frequency, power, as well as the exposure duration of mobile phones. There is also variability in the EEG recording details across the published papers. The most frequently utilized frequency is around 900 MHz, although a minimum of 450 MHz [42] and a maximum of up to 3500 MHz, around 5 GHz, were observed in studies [40,71]. The range of power, SAR, and amplitude variations associated with different frequencies exhibited considerable differences in the findings. Furthermore, concerning the duration of mobile EM exposure, a wide spectrum was observed, spanning from as short as 1 min [60] to as long as 8 h [93]. Pattnaik et al. [85] verified the effect of mobile EM exposure for both 2G and 3G, reporting an increase in theta, alpha, and beta activity while showing an increase in gamma band amplitude for 2G exposure in the eyes closed condition. Croft et al. [34] confirmed the effects of mobile EM exposure in 2G and 3G by just observing the increase in alpha band amplitude for mobile EM exposure 2G.

Some studies showed changes in certain electrodes, presenting a decrease in alpha band amplitude in some electrodes during the eyes closed condition [40].

Despite the missing information in certain columns of the table, it is important to note that no studies were disqualified or excluded.

Table 2 provides a comprehensive summary of the effects of mobile EM exposure on brain oscillations in the eyes opened condition. It shows the changes observed in brain oscillation parameters, including the amplitude of all EEG bands (Delta, Theta, Alpha, Beta, and Gamma), following mobile EM exposure. This table serves as a key resource for understanding the impact of mobile EM exposure on brain function and highlights significant findings from the included studies.

Table 2 shows that, out of 33 studies examining the effect of mobile EM exposure on all band power amplitudes using EEG in the eyes open recording conditions, four studies report an increase in delta band power amplitude, while three report a decrease. Five studies show an increase in theta band power, and three indicate a decrease. For alpha band power, fifteen studies show an increase and five studies report a reduction. Regarding beta band amplitude, eight studies indicate an increase and two indicate a decrease. For gamma-band power, three studies demonstrate an increase, whereas 30 studies show no effect. Overall, nine of the thirty-three studies suggest that mobile EM exposure does not influence brain oscillations.

Table 3 provides a comprehensive summary of the effects of mobile phone EM exposure on brain oscillations in the eyes closed conditions. Following mobile phone EM exposure, it verifies changes observed in brain oscillation parameters, including all bands’ amplitude power, delta, theta, alpha, beta, and gamma. This table summarizes the impact of mobile phone EM exposure on brain oscillations and highlights the key findings from the included studies.

As shown in Table 3, of the forty-eight studies investigating the effect of mobile EM exposure on all band power amplitudes using EEG in eyes closed recording conditions, six studies report an increase in delta band power amplitude, while three studies report a decrease. Regarding Theta band power, eight studies show an increase, and two show a decrease. For Alpha band power, twenty-four studies show an increase, and six studies report a reduction. Regarding Beta band amplitude, fourteen studies indicate an increase and four indicate a decrease. For gamma-band power, only one study proves an increase, whereas forty-seven studies show no effect. Overall, 13 of the 48 studies suggest that mobile EM exposure does not influence brain oscillations.

### 3.2. Effects of Mobile EM Exposure on Cortical Excitability

Table 4 provides a comprehensive summary of the effects of mobile EM exposure on cortical excitability. It details the changes observed in cortical excitability parameters following mobile EM exposure, including CSE, SICI, and ICF. This table shows the impact of mobile EM exposure on brain excitability and highlights significant findings from the included studies [21,22].

As shown in Table 4, in both included studies, ICF increased after exposure to mobile EM. Only one study [21] assessed SICI and showed a reduction in this outcome measure.

## 4. Discussion

This scoping review examines and catalogs the current literature, drawn from 80 studies, on the impact of mobile EM exposure on brain function. Of these, 78 studies investigated the effects of mobile EM exposure on brain oscillations using EEG, whereas only two studies examined CSE through TMS. The predominant findings indicated that after mobile EM exposure, studies observed an increase in the amplitude of brain oscillations across all frequency bands (delta, theta, alpha, beta, and gamma), especially during EEG recordings with eyes closed. Studies showed that these increases outweighed any reported decreases, with nearly 17 EEG studies being inconclusive. Additionally, one TMS study found that mobile EM exposure led to an increase in CSE and CCE within the ICF parameters.

This review focused on three key objectives: the primary aim of this review was to investigate the present literature regarding the impact of mobile EM exposure on changes in EEG band power amplitudes under both eyes opened and eyes closed conditions.

Regarding the first aim, almost all of the studies indicated that mobile EM exposure can induce changes (both increase and decrease) in EEG band power amplitude (Table 2 and Table 3). We aimed to assess the effects of mobile EM exposure on the amplitude of EEG band power during the eyes opened condition in healthy participants (Table 2). The 33 of the studies examining the effect of mobile EM exposure on all band power amplitudes via EEG in eyes opened conditions observed increases and decreases, as well as no effect in all band power amplitudes, although the number of studies showing an increase [52] was higher than those showing a decrease [39] and no effect [45].

Also, we planned to evaluate how mobile EM exposure affects all EEG band power amplitudes during the eyes closed condition in healthy participants (Table 3). Across the 48 studies examining this effect, we observed varying trends in band power amplitudes, including increases and decreases, without any significant effect. The evaluated results confirmed that the number of studies reporting increases [85] tended to exceed those reporting decreases [80] and had no effect [68].

Our investigation revealed a lack of consensus on the observed changes across all EEG bands. This discrepancy can be attributed to methodological differences among the included studies. Specifically, different generations of mobile telecommunication networks (3G, 4G, and 5G) were used across the studies. Variations in the duration of exposure, as well as differences in the characteristics of the hardware and software used, including diverse parameters for filtering EEG signals, contributed to the inconsistencies. These factors combined have led to inconclusive results regarding the effect of mobile EM exposure on the amplitude of EEG bands during the eyes opened condition and eyes closed condition in healthy participants. Therefore, we conclude that the effect of mobile EM exposure on the EEG bands’ power amplitude remains inconclusive.

The key question we need to address is which mechanisms allow mobile EM exposure to influence brain oscillations. Mobile EM exposure can cause localized heating in the brain tissues, which can alter neuronal activity and potentially affect the amplitude and frequency of EEG signals [105,106]. This thermal effect may also lead to changes in blood flow within the brain, influencing neural activity and thus EEG patterns [106,107,108,109,110]. Beyond thermal effects, mobile EM exposure might directly interfere with the electrical activity of neurons, causing changes in normal oscillatory patterns observed in EEG [111,112,113,114,115]. Exposure to mobile EM exposure may also affect ion channel behavior in neuronal membranes, impacting neuronal activity and synaptic transmission [116,117,118]. Mobile EM exposure can induce oxidative stress in neural tissues, leading to alterations in neural function and EEG signals [119,120,121,122,123,124]. These combined effects underscore the multifaceted ways in which mobile EM exposure can influence brain oscillations. However, the exact nature and extent of these effects remain an area for further research due to varying study methodologies and individual differences.

The second aim of this scoping review was to examine TMS studies in the existing literature regarding the effects of mobile EM exposure on changes in CSE and its underlying mechanisms through paired-pulse paradigms such as SICI, LICI, and ICF. Only two articles were included (Table 4). In both studies, ICF increased, which appears to be primarily due to enhanced facilitatory mechanisms and reduced inhibitory mechanisms. The limited scope, with only two studies on mobile EM exposure effects on CSE and CCE, restricts the ability to review both supporting and opposing evidence in this field, thus hindering the opportunity to draw definitive conclusions.

The exact mechanisms by which mobile EM exposure affects cortical excitability in the brain are not yet fully understood, and ongoing scientific research is continuing to explore this area. However, several mechanisms have been proposed. Mobile devices emit non-ionizing EM fields within the microwave range, which can penetrate tissues and potentially induce heating. This slight thermal effect can impact brain tissue, leading to alterations in CSE [125,126,127,128,129,130]. Various non-thermal mechanisms involving the interaction of EMFs with biological processes at the cellular level can explain the effects. Mobile EM exposure may influence tissues and cells, resulting in an effect on the release of neurotransmitters in the brain. Given the critical role of neurotransmitters in neural signalling, any modifications in their functionality could lead to changes in CSE [131,132]. Exposure to mobile EM exposure may also have been associated with alterations in gene expression within the brain. Changes in patterns of gene expression may lead to modifications in neural function, potentially influencing CSE [108,111,133,134,135,136,137,138,139,140]. Although the effects of mobile phone EM exposure on cellular biological pathways such as Ion channel mechanisms and Oxidative stress have been mentioned earlier in the discussion section, several in vitro animal studies have specifically examined the cellular and molecular pathways within the neural system [137,141,142,143].

The third aim of this scoping review was to identify gaps in the literature and recommend future research directions. While extensive research has explored the global impact of EMR energy and the thermal and non-thermal effects of previous mobile phone technologies like 3G and 4G on brain tissues, a comprehensive evaluation of the non-thermal effects of mobile EM exposure is still lacking. Consequently, an urgent imperative exists for further research, with a particular focus on 5G technology, which represents a more advanced and practical approach compared to the previous mobile phone generations. Although several studies have explored the consequences of earlier generations of mobile phones on human health and organizations like the International Commission on Non-Ionizing exposure Protection (ICNIRP) have worked to enhance safety guidelines [7,144], it is clear that significant knowledge gaps exist in our understanding of the potential impacts of mobile EM exposure.

The existing literature on the effects of 5G mmWave mobile EM exposure suggests a number of areas where further research may be beneficial. One notable gap is the limited number of studies examining how 5G mmWave exposure may affect brain oscillations and excitability. Moreover, there are insufficient data on how mobile phone exposure affects brain function with regard to factors such as gender, age, and the type of mobile phone used, including GSM mobile phones and smartphones, as well as the placement and proximity of these devices to the brain. Another issue is that many studies do not provide detailed methodologies, such as pre-determined effect sizes based on power and sample size considerations. Important information such as frequency, SAR, power, power density, mobile phone brands, and the distance of the device from the brain is often not included in these studies. Studies using EEG often fail to provide adequate details about the EEG channel systems, including the number and placement of electrodes, the frequencies analyzed, and recording conditions such as whether the subjects’ eyes were open or closed during the EEG sessions. Moreover, there is a scarcity of data on brain excitability assessments using TMS, a reliable method for evaluating CSE. This includes the use of single-pulse MEP and paired-pulse TMS to investigate SICI, LICI, and ICF as potential mechanisms influencing changes in CSE. Addressing these gaps through well-designed studies would be crucial for improving our understanding of the potential biological effects of 5G technology on brain function and excitability.

This scoping review highlights the field by addressing inconsistencies in prior studies regarding the effects of RF exposure on brain oscillations. It demonstrates consistent increases in alpha and beta power amplitudes, particularly in eyes closed conditions, which underscores the implications for understanding cognitive states and neural process synchronization [61,84]. Previous reviews just focused on 3G/4G mobile phone EM exposure; however, this review highlights the urgent need to investigate the long-term effects of 5G on the brain, emphasizing that addressing this gap is essential for conducting precise safety guidelines [113,145]. The review also calls for the need to account for demographic variables, particularly age and gender, when grouping participants in research, which likely influences responses to RF exposure. Some studies also acknowledge this [146,147,148], but many research studies still fail to incorporate sufficient age and gender-specific analyses. Additionally, it presents the use of TMS and EEG as non-invasive methods to verify RF exposure effects with direct neurophysiological outcomes through cortical excitability [22] assessments and brain oscillation [49].

To provide a clearer overview of the research evolution and highlight the existing gaps, Table 5 summarizes the frequency bands used across mobile generations, their main applications, existing research, and the identified research gaps [149].

## 5. Limitations

The findings of the current scoping review should be interpreted carefully and with caution, given some limitations. The first concern is the very limited number of TMS studies in the literature to assess CSE and CCE, considering comprehensive protocols, which limits judgment and identification of the existing TMS findings. The second limitation is the excessive diversity in assessment parameters across different EEG studies, which prevents the possibility of leading a meta-analysis, specifically when regarding the effects of mobile EM exposure on brain oscillations. Additionally, since only English-language papers were included, some relevant studies in other languages may have been overlooked. Other concerns, such as imprecise volunteer selection regarding age and gender and the lack of pre-defined effect sizes based on power and sample size considerations, may affect the overall strength and generalizability of the results.

## 6. Conclusions and Future Work

Our literature review indicates that mobile EM exposure can induce changes in brain oscillations, primarily increasing the amplitude of alpha, beta, theta, and delta bands, particularly in the eyes closed condition. This review suggests that mobile EM exposure may enhance CSE due to strengthened facilitator mechanisms and weakened inhibitory mechanisms. Despite extensive research over the years, several gaps remain in the literature, which require further investigation, particularly regarding the effects of 5G as an emerging technology. This is linked to the non-thermal effect even at exposure levels below current thermal thresholds. These findings underscore the need to revise SAR limits and to incorporate neurophysiological EEG and TMS metrics as non-invasive measures. Furthermore, the advent of 5G technology, with its use of higher frequency mmWave bands (>24 GHz), demands urgent investigation into potential unique tissue interactions and indirect neural impacts, which warrants precautionary measures until robust safety data are available. The conclusions of this review may be judged as consistent with the evidence. However, they should be treated with caution due to key limitations, such as limited TMS studies (only two), which resulted in a lack of data and some methodological and technical problems in EEG studies, as mentioned in the discussion and limitations section. Additionally, the lack of research verifying 5G frequencies above 6 GHz prevents definitive claims.

The following recommendations are suggested for future research in this field: (1) Conduct more comprehensive examinations of the effects of 5G mobile EM exposure on brain oscillations and brain excitability. This requires additional experimental research, including exposure systems operating at 5G frequencies and post-exposure assessments. (2) Study the impact of age factors on brain structure and physiology by assessing mobile EM exposure effects on both young and older adults to better understand age-related differences. (3) Investigate potential gender effects of mobile EM exposure on brain structure and function. (4) Enhance the precision of future research by refining methodological aspects, from data acquisition to result analysis, in EEG and TMS studies. This includes careful attention to study design, optimal sample size determination, and comprehensive study setup, all contributing to improved accuracy in future investigations. (5) Standardize exposure parameters (frequency, SAR, distance from the head), sham/control conditions (double-blind protocols), and reporting EEG/TMS technical details (electrode placement, filtering) in EEG and TMS protocols, which are critical updates to international EM exposure guidelines.

## Figures and Tables

**Table 1:** Keywords and search terms used in this review.

	Keywords Inclusion	Keywords Exclusion
**Population**	Healthy individuals, Adults, Humans, Healthy people, Able-bodied adults	Pathological disorders, Animal studies
**Concept**	EM radiation, EMR, Mobile phone radiation, mobile EM, 3G/4G/5G networks, Cell phone radiation Mobile phone frequency, MPF, Radiofrequency radiation, RFR, Low-level radiation, Wireless emissions, Wireless radiation, Mobile device radiation, Cellular radiation, Radio wave exposure, Mobile phone generations	Cognitive function
**Context**	Brain function, Brain activity, Brain oscillation, Neural oscillations, Electroencephalography, EEG, Transcranial magnetic stimulation, TMS, Brain wave, Brainwave activity, Cortical excitability, Corticospinal excitability, CSE	Ionizing radiation, Non-invasive brain stimulation techniques such as Transcranial Direct Current Stimulation (tDCS), Transcranial Alternating Current Stimulation (tACS), Transcranial random noise stimulation (tRNS), Transcranial pulsed current stimulation (tPCS)

**Table 2:** Experimental EEG studies investigating the effects of mobile phone EM exposure on brain oscillations (eyes opened).

Study	Participants (Gender)/ Age Range (Mean)	Mobile Phone Specification (Frequency/Power/SAR)	Exposure Time (min)	Study Design (Crossover)/Blinding		Number of Channels and/or Electrodes	Effects on Band Amplitude				
				Single-Blind	Double-Blind		Delta	Theta	Alpha	Beta	Gamma
[19]	24 (16 M, 8 F)/19–48 Y, 27.5 Y	900–1800 MHz/250 mW	20	*√*	-	19	↓	↑	↑	↑	-
[25]	120 (46 M, 74 F)/18–69 Y	895 MHz/2 W, 0.674 W/kg	30	-	*√*	58	-	-	↑	-	-
[26]	34 F/20 ± 3 Y	1920 to 2170 MHz/-	20	-	*√*	32	-	-	↓	-	-
[27]	10 M (adults), 10 children/12 Y	900 MHz/-	10	-	*√*	16	↑	-	-	-	-
[28]	36 (18 M, 18 F)/18–52 Y	920 MHz/2 W/kg	30	-	*√*	19	-	-	↑	-	-
[29]	36 M/23.6 Y	902 MHz/0.25 W	40	-	*√*	5	-	-	↑	-	-
[30]	10 M/-	900 MHz, 2 W/kg	30	*√*	-	12	↑	-	↑	-	-
[31]	15 F/21–30 Y	2.476 GHz/80 μW/kg	5 & 15	-	-	8/21	-	↑	↑	↑	-
[32]	72 (37 M, 35 F)/24.5 Y	Nokia 3110/-	20	-	*√*	19	-	-	↓	-	-
[33]	72 (37 M, 35 F)/24.5 Y	900 MHz/1.95 W/kg	20	-	*√*	19	-	-	↑	-	-
[34]	42 (21 M, 21 F)/19–40 Y	894.6 MHz (2G) 250 mW/Vs 1900 MHz (3G)/1.7 W/kg	50	-	*√*	61	-	-	↑	-	-
[35]	24 M/19–25 Y	900 MHz/1 W/kg	30	-	*√*	-	-	-	-	-	-
[20]	26 (13 M, 13 F)/23.5 Y	900 MHz/-	26	-	*√*	29	-	-	-	-	-
[36]	22 (12 M, 10 F)/11–13 Y	900 MHz/-	30	-	*√*	-	-	-	-	-	-
[37]	21 (11 M, 10 F)/25.1 Y	900 MHz/0.49 W/kg	25.5	-	*√*	74	-	-	-	-	-
[38]	20 (M, F)/18–28 Y	450–2500 MHz/2 W	5	-	*√*	16	↓	↓	↓	↑	-
[39]	21 (11 M, 10 F)/25.1 ± 3.6 Y	900 MHz/0.49 W/kg	25	-	*√*	74	↓	↓	-	↓	-
[40]	34 (17 M, 17 F)/26.6 ± 4.7 Y	3.5 GHz/5G Hz/ 0.037 ± 0.11 mW/kg	25.5	-	-	64	-	-	-	-	-
[41]	36 (18 M, 18 F)/18–52 Y	920 MHz/2 W/kg	30	-	*√*	19	-	-	↑	-	-
[42]	23 (12 M, 11 F)/21–24 Y	450 MHz/0.16 mW/cm^2^, 0.35 W/kg	10 (1 min ON/OFF)	-	*√*	9	-	-	↑	↑	-
[43]	77/-	450 MHz	-	-	*√*	-	-	-	-	↓	↓
[44]	8 (6 M, 2 F)/28 Y	COMSOL 2004	6	*√*	-	4/19	-	-	↑	↑	-
[45]	20 M/19–36 Y	900-MHz/10 W/m^2^	480	-	-	2	-	-	-	-	-
[46]	13 M/21–27 Y	916.2 MHz/2.8 W	-	*√*	-	30	-	-	-	↑	-
[47]	31 F/26.7 Y	5 Hz–3 GHz or 1.9291 to 1.9397 GHz/0.69 W/kg	15	*√*	-	12	-	-	↑	↑	↑
[48]	5/-	900 MHz, 1800 MHz/-	1	-	*√*	32	↑	↓	↓	↑	-
[49]	20 (9 M, 11 F)/-	GCM: Nokia 105 vs. SP: Huawei P8lite	5	-	*√*	5	-	↑	↑	-	-
[50]	36 (16 M, 20 F)/19–28 Y	3G/1.75 W/kg	20	-	*√*	2/3	-	-	-	-	↑
[51]	35 (18 M, 17 F)/39.8 Y	-	5	-	*√*	21	-	-	-	-	-
[52]	48 (21 M, 27 F)/18–44 Y	884 MHz	3	-	*√*	8	↑	↑	↑	-	-
[53]	29 (19 M, 10 F)/18–40 Y	Android 2500 mAh battery	5	-	-	128	-	↑	↑	-	↑
[54]	25 (10 M, 15 F)/23.3 Y	2.4 GHz/99.22 mW/kg	60	-	*√*	13/5	-	-	-	-	-
[55]	17 (8 M, 9 F)/21.76 Y	1947 MHz/1.75 W/kg	30	-	*√*	3	-	-	-	-	-

Note: M: Male; F: Female; Y: years old; F; frequency; SAR; specific absorption rate; MHz: Megahertz; GHz: Gigahertz; EO: Eyes opened; EC: Eyes closed, channels; GCM: Google Cloud Messaging Smartphone; 2G: 2nd generation; 3G: 3rd generation; 4G: 4th generation; ↑ = increased; ↓ = decreased; - = no significant changes; *√* = yes.

**Table 3:** Experimental EEG studies investigating the effects of mobile phone EM exposure on brain oscillations (eyes closed).

Study	Participants (Gender)/ Age Range (Mean)	Mobile Phone Specification (Frequency/Power/SAR)	Exposure Time (min/s)	Study Design (Crossover)/Blinding		Number of Channels and/or Electrodes	Effects on Band Amplitude				
				Single-Blind	Double-Blind		Delta	Theta	Alpha	Beta	Gamma
[56]	20 (10 M, 10 F)/22–31 Y	902.40 MHz/0.25 W	45	-	*√*	5	-	-	↑	-	-
[57]	397 (50.9% F)/>18 Y	900 MHz–1800 MHz/	For 5 nights	-	*√*	-	-	-	-	-	-
[58]	34 M/20–30 Y	2.45 GHz/6.4 mW/kg	Every six minutes for 5 nights	-	*√*	19	↓	-	↓	↓	-
[59]	19 (10 M, 9 F)/M: 28–48 Y, F: 32–57 Y	900, 1800 MHz/1–2 W	20	*√*	-	21	↑	-	-	-	-
[60]	13 (4 M, 9 F)/21–30 Y	450 MHz/0.16 mW/cm^2^	1	-	*√*	9	-	-	↑	↑	-
[61]	34 (16 M, 18 F)	450 MHz/0.16 mW/cm_2_	10 (1 min ON/OFF)	-	*√*	9	-	-	↑	↑	-
[62]	34 M/21–35 Y	900 MHz/0.05 mW/cm_2_	3.5	-	-	-	-	-	-	-	-
[63]	14 (7 M, 7 F)/21–24 Y	450 Hz/0.16 mW/cm^2^	10 (1 min ON/OFF)	*√*	*√*	8/9	-	-	↑	↑	-
[64]	15 (8 M, 7 F)/23–32 Y	450-MHz/0.303 W/kg	10 (1 min ON/OFF)	-	-	9/19	-	-	↑	↑	-
[65]	28/21–30 Y	450 Hz/0.303 W/kg	10 (1 min ON/OFF)	-	*√*	9	-	-	-	↑	-
[66]	16 M	900 MHz/1 W/kg	30	*√*	-	-	-	-	↑	-	-
[67]	30/-	450 to 2500 MHz	5	-	*√*	2	↑	-	↓	↑	-
[68]	10 M/25.2 Y	2.573 GHz/-	30	-	*√*	32	-	-	-	-	-
[69]	18 (12 M, 6 F)/19–35 Y	800–3500 MHz/2.18–2.36 W/kg	30	-	*√*	8	↓	↑	↑	-	-
[70]	15 (8 M, 7 F)/21–24 Y	450 MHz/0.16 mW/cm^2^	10	-	*√*	19	-	-	↑	-	-
[71]	38/20–36 Y	5000 kHz–3 GHz 2.0 W/kg	30	-	*√*	128	-	-	-	-	-
[72]	18F/39 Y	450 MHz/0.9 mW/cm^2^	30	*√*	-	19	-	↑	-	↑	-
[73]	15 (8 M, 7 F)/21–24 Y	450 MHz/0.16 mW/cm^2^	5 (1 min ON/OFF)	-	*√*	9	-	-	↑	↑	-
[74]	30 M/20–30 Y	100 kHz–3 GHz/1.5 W/kg	12,960	-	*√*	19	-	-	-	-	-
[75]	10 (5 M, 5 F) 18–30 Y	900 MHz/250 mW	5	*√*	-	6	-	-	↓	↓	-
[76]	5/- 18–27 Y	900–1800 MHz/-	>10 min	-	*√*	19	↓	↓	↑	↓	-
[77]	10 M/25–36 Y	902.40 MHz/0.25 W	45	-	*√*	19	-	-	↑	-	-
[78]	16 (7 M, 9 F)/47–84 Y	902.40 MHz/0.5 W/kg	45	-	*√*	19	-	-	↑	-	-
[79]	20 M/22–37 Y	900 MHz/	-	*√*	-	6	-	-	-	-	-
[80]	45/-	-/0.69 W/kg	5	-	-	2/4	-	-	↓	↓	-
[81]	66 (30 M, 36 F)/19–24 Y	450 MHz/0.303 W/kg 0.16 mW/cm^2^	20 and 40	-	*√*	9/9	-	-	↑	↑	-
[82]	28 (13 M, 15 F)/20–27 Y	450 MHz/0.16 mW/cm^2^/0.303 W/kg	10 cycle every odd min	-	*√*	8/9	-	-	↑	-	-
[83]	52 (25 M, 27 F)/19–76 Y	27.12 MHz/4 mW/kg	15	-	*√*	8	↑	-	-	-	-
[84]	25 M/30.2 ± 2.7 Y	2.61 GHz	30	-	*√*	19	-	-	↑	↑	-
[85]	75 (57 M, 18 F)/22.2 Y	Nokia, Samsung, Panasonic, and Motorola, 2G,3G/0.67 and 1.14 W/kg	5	-	-	16/21	-	↑	↑	↑	↑
[86]	16 M/20–25 Y	900 MHz/-	30 pulls	*√*	-	-	-	-	↑	-	-
[34]	42 (21 M, 21 F)/19–40 Y	894.6 MHz (2G) 250 mW/Vs 1900 MHz (3G)/1.7 W/kg	50	-	*√*	61	-	-	-	-	-
[35]	24 M/19–25 Y	900 MHz/1 W/kg	30	-	*√*	-	-	-	↑	-	-
[20]	26 (13 M, 13 F)/23.5 Y	900 MHz/-	26	-	*√*	29	-	-	↓	-	-
[36]	22 (12 M, 10 F)/11–13 Y	900 MHz/-	30	-	*√*	-	-	-	-	-	-
[87]	60 M/- 20 ± 25 Y	900 MHz/o 2.2W	30	-	*√*	9	-	-	↑	↑	-
[38]	20 (M, F)/18–28 Y	450–2500 MHz/2 W	5	-	*√*	16	↓	↓	↑	↑	-
[37]	21 (11 M, 10 F)/25.1 Y	900 MHz/0.49 W/kg	25/5	-	*√*	74	-	-	-	-	-
[40]	34 (17 M, 17 F)/26.6 ± 4.7 Y	3.5 GHz/5G Hz/0.037 ± 0.11 mW/kg	25/5	-	-	64	↑	↑	↓	-	-
[39]	21 (11 M, 10 F)/25.1 ± 3.6 Y	900 MHz/0.49 W/kg	25	-	*√*	74	-	↑	-	-	-
[41]	36 (18 M, 18 F)/18–52 Y	920 MHz/2W/kg	30	-	*√*	19	-	-	-	-	-
[88]	36 (18 M, 18 F)	902.4 MHz/-	15	*√*	-	16	-	-	↑	↑	-
[89]	16 M/18–21 Y	900 MHz/10 W/kg	10 (1 min ON/OFF)	-	*√*	10/12	↑	↑	-	-	-
[90]	20 (7 M, 13 F)/20–51 Y	894.6 MHz/2 W	30	-	*√*	2/4	-	-	↑	-	-
[91]	25 M/20–26 Y	900 MHz/2 W/kg	30	-	*√*	2	↑	↑	↑	-	-
[92]	12/24.6	1.8 GHz/-	5	*√*	-	9	-	↑	↑	-	-
[93]	30 M/24.1 ± 2.9 Y	2.45 GHz/6.4 mW/kg	480	-	*√*	19	-	-	-	-	-
[94]	15 M/20–35 Y	900,1950 MHz/-	2 and 6	-	*√*	19	-	-	-	-	-
[95]	50 (27 M, 23 F)/18–60 Y	914 MHz 0.19 ± 0.03 W/kg	30	-	*√*	2	-	-	↑	-	-

Note: M: Male; F: Female; Y: years old; Frequency; SAR: specific absorption rate; MHz: Megahertz; GHz: Gigahertz; W/kg: Watts per Kilogram; cm^2^: centimetres squared; ↑ = increased; ↓ = decreased; - = no significant changes; *√* = yes.

**Table 4:** TMS studies investigate the effects of mobile phone exposure on CSE, SICI, and ICF.

Study	Participants Gender Age Range (Mean)	Mobile Phone Specification (Frequency/Power/SAR)	Exposure Time/min	Study Design (Crossover)/Blinding		TMS Characteristics	Observed Effects			
				Single-Blind	Double-Blind		CSE	ICF	SICI	LICI
[22]	10 (5 M, 5 F)/22–51 Y	800 MHz/270 mW	30	-	*√*	200 (The Magstim Co., Ltd., Whitland, UK)	-	↑	N/S	N/S
[21]	15 F/20–36 Y	902.40 MHz/2 W	45	-	*√*	200 (The Magstim Co., Dyfed, UK)	-	↑	↓	N/S

Note: M: Male; F: Female; Y: years old; F; frequency; SAR; specific absorption rate; MHz: Megahertz; W: watt; ↑ = increased; ↓ = decreased; - = no significant changes; *√* = yes; N/S = Not Studied.

**Table 5:** Mobile technology, research status, and gaps in the literature.

Technology	Frequency Band	Main Applications	Existing Research	Research Gaps
1G/2G	~800–900 MHz	voice communications	Extensive studies on SAR (Specific Absorption Rate), health risk assessments	Limited newer studies reassessing low-level long-term exposure
3G	~1.8–2.1 GHz	providing access to the Internet on laptops and mobile devices	Significant research on RF biological effects and epidemiological studies	Need for updated risk assessments based on modern usage patterns
4G	~2–2.6 GHz (sometimes up to 3.5 GHz)	video conferencing to gaming services, HD mobile television	Good coverage of thermal and some non-thermal effects, SAR compliance	Gaps in chronic exposure studies, particularly for high-data applications
5G (Sub-6 GHz)	~3.3–6 GHz	Enhanced mobile broadband, IoT, smart cities	Early studies, focusing mainly on compliance testing and exposure modeling	Need more biological studies on non-thermal effects, long-term exposure
5G (mmWave)	24–28 GHz, 37–40 GHz, 60 GHz	Ultra-fast broadband, high-capacity urban networks, AR/VR	Very limited; few experimental biological studies, mainly simulations	Major gaps in biological effects, tissue interaction, long-term health risks, thermal load at skin/tissue interface

## Data Availability

The data supporting this study’s findings are available from the corresponding author upon reasonable request.
